# *Macrobrachium rosenbergii* Genome Editing Breeding with CRISPR–Cas Nucleases, Base Editors, and Prime Editors

**DOI:** 10.3390/ani15152161

**Published:** 2025-07-22

**Authors:** Guo Li, Xinzhi Zhou, Guanglin Zhu, Yingjia Pan, Junjun Yan, Jilun Meng, Tiantian Ye, Yaxian Cheng, Cui Liu, Zhimin Gu

**Affiliations:** 1 Xianghu Laboratory, Hangzhou 311231, China; liguo@xhlab.ac.cn (G.L.); zhuguanglin@tju.edu.cn (G.Z.); yanjunjun@xhlab.ac.cn (J.Y.); mengjilun@xhlab.ac.cn (J.M.); yetiantian@xhlab.ac.cn (T.Y.); yxcheng@zju.edu.cn (Y.C.); liucui@xhlab.ac.cn (C.L.); 2 College of Chemical and Biological Engineering, Zhejiang University, Hangzhou 310027, China; zhouxinzhi@zju.edu.cn (X.Z.); yj_pan@zju.edu.cn (Y.P.); 3 State Key Laboratory of Animal Biotech Breeding, China Agricultural University, Beijing 100193, China

**Keywords:** genome editing, base editors, prime editors, *M. rosenbergii*, breeding

## Abstract

This review focuses on CRISPR genome editing technology, particularly its application in the study of *Macrobrachium rosenbergii* (*M. rosenbergii*). It first elaborates on the basic principles and mechanisms of CRISPR–Cas9 technology, base editors, and prime editors. Then, it explores the application of this technology in *M. rosenbergii* breeding, including improving growth rate, enhancing disease resistance, and sex control. Additionally, it introduces the progress of genome editing technology in *M. rosenbergii*, epidemiology and pathogenesis, diagnostic techniques, analyzes the opportunities and challenges it faces, reviews the historical evolution, and looks ahead to future development directions. CRISPR technology has brought new opportunities to the research and industrial development of *M. rosenbergii*, but it also needs to address numerous technical and safety challenges.

## Simple Summary

The giant freshwater prawn (*Macrobrachium rosenbergii*) is a key species in global aquaculture, but challenges like slow growth, disease susceptibility, and aggressive behavior limit productivity. This review explores how advanced genome editing tools (CRISPR–Cas9, base editors and prime editors) can improve breeding by precisely modifying genes to enhance growth, disease resistance, and sex control. These technologies allow targeted changes without disrupting other genetic functions, offering faster and more efficient improvements than traditional breeding. However, challenges remain, including delivery methods, off-target effects, and regulatory concerns. Successful application could revolutionize aquaculture by creating hardier, faster-growing prawns, thereby boosting food security and sustainability. Future research aims to refine these tools for safe, large-scale use in *M. rosenbergii* and other aquatic species.

## 1. Introduction

The giant freshwater prawn *M. rosenbergii* is a cornerstone of global aquaculture, particularly in Asia, due to its high economic value and adaptability to farming systems [1,2]. However, challenges such as aggressive behaviors in high-density cultures, disease susceptibility, and slow genetic improvement through traditional breeding have spurred interest in advanced genome editing technologies like CRISPR–Cas nucleases [3,4], base editors (BE) [5], and prime editors (PE) [6,7,8,9] to enhance productivity and resilience.

Prior to the advent of CRISPR-based systems, genetic manipulation in *M. rosenbergii* mainly relied on low-throughput techniques with significant limitations. Traditional methods, such as transgenesis by electroporation or viral vectors (e.g., baculovirus), enabled the delivery of foreign genes like GFP reporters into embryos but resulted in random integration rates below 5% and only transient expression [10]. Similarly, genome editing tools like TALENs and ZFNs were employed to disrupt genes such as the sex-determination factor MrIAG, but their reliance on complex protein engineering led to low mutagenesis efficiencies (1–3%) and poor scalability [11]. RNA interference (RNAi) allowed for targeted gene knockdown, exemplified by studies on the MrVg gene involved in vitellogenesis [12], but its effects were partial and reversible [13].

However, fundamental challenges hindered the broader application of these methods. TALENs and ZFNs required custom protein design, increasing off-target risks and reducing precision compared to the modular guide RNA system of CRISPR. Microinjection efficiency was also low, with success rates below 20% due to the species’ hardened chorion [14], a barrier later addressed by CRISPR-RNP delivery, which achieved efficiencies of 30–60% [2]. Moreover, scalability remained limited: multiplex gene editing was unfeasible with ZFNs and TALENs, while CRISPR enables the simultaneous targeting of multiple genes, including those involved in growth and immune regulation [15].

**Figure 1:**
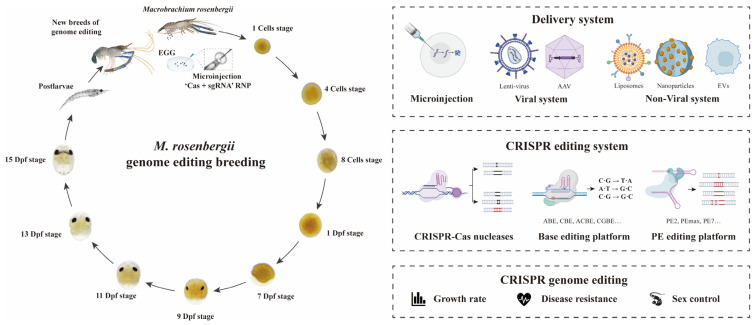
Genome editing applications of *M. rosenbergii* by using Cas nuclease, base editor, and prime editor CRISPR technologies. The graphic abstract illustrates the application of CRISPR-based genome editing tools, including Cas nucleases (for gene knockouts), base editors (enabling precise single base substitutions), and prime editors (supporting targeted insertions, deletions, and replacements) in *M. rosenbergii*. The schematic highlights their roles in functional gene studies, trait enhancement (e.g., growth, disease resistance), and the development of non-injection delivery systems to advance aquaculture breeding programs.

CRISPR–Cas9 has emerged as a transformative tool in crustacean genomics, enabling precise gene knockouts and functional studies. For instance, pioneering work targeting developmental genes like *Pax6* (critical for eye development) and cofilin (involved in actin dynamics) in *M. rosenbergii* embryos demonstrated successful gene editing with Cas9 ribonucleoprotein (RNP) complexes, achieving higher efficiency in embryos than in primary cell cultures [2]. These studies highlight the feasibility of CRISPR for disrupting genes linked to growth, behavior, or the reproduction of *M. rosenbergii* by using emerging CRISPR technologies, such as Cas nucleases, base editors, and prime editors, to develop new breeds, and examines the challenges associated with genome editing in this species, including the development of non-injection delivery systems (Figure 1).

## 2. CRISPR–Cas9 Genome Editing Technologies

The CRISPR–Cas9 system originates from the adaptive immune defense mechanism of bacteria, which can identify and cleave exogenous DNA from invading phages or plasmids. The system is primarily composed of the Cas9 nuclease and a guide RNA (gRNA) [16]. The gRNA contains a sequence complementary to the target DNA, guiding the Cas9 protein to bind precisely to the target DNA site. Subsequently, the Cas9 nuclease cleaves the double-stranded DNA at a specific position upstream of the PAM (protospacer adjacent motif) sequence, creating a double-strand break (DSB). Cells repair DSBs mainly through two pathways: non-homologous end-joining (NHEJ) and homology-directed repair (HDR) [17]. The NHEJ repair process is error-prone and often results in insertion or deletion mutations, which can be used for gene knockout. In contrast, HDR uses a homologous template for precise repair when available, enabling gene knockin or replacement. For instance, in studying specific gene functions, the NHEJ pathway can be employed to induce frameshift mutations, disrupting gene function and thereby exploring the gene’s role in biological processes. The emergence of this technology has significantly advanced fields such as gene function research and gene therapy [18].

## 3. Base Editing Technologies

Base editors (BEs) are novel genome editing tools derived from the CRISPR–Cas9 system that enable the conversion of specific bases without inducing double-strand breaks. They are primarily categorized into two types: cytosine base editors (CBEs) and adenine base editors (ABEs). CBEs can convert C•G base pairs to T•A base pairs, while ABEs can convert A•T base pairs to G•C base pairs [19]. The efficiency and precision of CBEs and ABEs make them highly promising for applications in genome editing. For example, CBEs have been used for precise genome editing in plants to improve crop traits [20]. Additionally, ABEs have demonstrated efficient editing capabilities in mammalian cells, enabling precise gene editing without introducing double-strand breaks [21]. However, base editors also face challenges, such as limited types of targetable edits and potential off-target effects [22]. Studies have shown that CBEs and ABEs may cause non-target DNA editing and RNA deamination, which could lead to unintended genomic changes [23]. Therefore, developing base editor variants with higher specificity and lower off-target effects is a key direction for current research [24,25,26]. Besides ABE and CBE, researchers have developed a variety of base editors capable of diverse editing functions, including gBE (mediating G > C and G > T editing) [27], TBE (targeting T > C and T > G substitutions) [28,29], and AX/YBE (enabling A > C and A > T modifications) [30,31]. Currently, researchers have developed a comprehensive suite of base editors capable of precise single-base editing for functional genes (Table 1).

Furthermore, base editors have been applied to edit disease-related genes in animal models. For instance, ABEs have been used to achieve efficient gene editing in mouse models, successfully mimicking human disease-associated gene mutations [66]. These advancements offer new possibilities for gene therapy and hold promise for treating various genetic diseases [67]. In summary, base editors, as an emerging genome editing tool, have broad application prospects and research value. With continuous technological development and optimization, base editors are expected to play a greater role in basic research and clinical applications.

## 4. Prime Editing Technologies

Prime editing is a novel and precise genome editing technology that enables a variety of DNA sequence modifications, such as base substitutions, small insertions, and deletions, without relying on double-strand breaks or donor DNA templates [6]. This technology is composed of a modified Cas9 nickase (nCas9) fused to a reverse transcriptase (RT) and is guided by a specially designed prime editing guide RNA (pegRNA). The pegRNA contains a sequence complementary to the target DNA and carries a reverse transcription template that guides the synthesis of a new DNA strand with the desired edit. During the editing process, nCas9, guided by the pegRNA, creates a nick in one strand of the target DNA. The exposed 3′ end serves as a primer, and the reverse transcriptase synthesizes a new strand using the pegRNA’s template. Subsequently, cellular repair mechanisms integrate the new strand into the genome, completing the precise edit. In recent years, prime editing has been widely applied in various cell types, showing high editing efficiency and low byproduct formation [68].

By optimizing the structure of pegRNAs and editing proteins, researchers have developed enhanced prime editing systems, which significantly improve editing efficiency. Researchers have developed a diverse suite of prime editors capable of programmable ‘search-and-replace’ genome editing, enabling single-base substitutions, small insertions, deletions, and precise insertion of large DNA fragments (Table 2). The applications of prime editing are not limited to human cells but have also been successfully applied in plant genome editing [69]. By introducing prime editing into plants, researchers can achieve precise genomic modifications, providing new tools for crop trait improvement and biological research. Furthermore, prime editing has been used in bacterial genome engineering, where inhibiting DNA exonucleases has significantly improved editing efficiency [70]. In clinical applications, prime editing is considered to have great potential for repairing mutations associated with human genetic diseases [71]. Despite current challenges, such as optimizing editing efficiency and delivery strategies, prime editing is expected to play a significant role in gene therapy as the technology continues to develop [72]. In summary, prime editing, as a revolutionary genome editing tool, is continuously expanding its scope and capabilities. Through ongoing technological optimization and innovation, prime editing is expected to play a greater role in basic research and clinical applications, bringing new opportunities for human health and agricultural development.

## 5. CRISPR Technologies in *M. rosenbergii* Breeding

Early genetic studies on *M. rosenbergii* laid the foundation for understanding its genetic makeup. Research on hemocyanin isoform 2 (MrHc2) not only characterized its structure but also revealed its role in the innate immune response to MrNV [80]. The identification of microsatellite DNA markers in *M. rosenbergii* provided a tool for genetic analysis. These markers, with the number of alleles per locus ranging from 3 to 16, and observed heterozygosities between 0.22 and 0.71, are useful for population genetic studies and the conservation of wild and cultured stocks [81]. These early genetic findings set the stage for the development of more advanced genome editing techniques in *M. rosenbergii*.

## 6. Genome Editing for Enhanced Growth Rate in *M. rosenbergii* Breeding

The application of CRISPR genome editing technology to enhance the growth rate in *M. rosenbergii* breeding is of great significance. The advent of CRISPR technology has revolutionized genome editing, enabling precise modifications within organisms. This technology, widely used in plant breeding, also shows tremendous potential in aquaculture. The application of CRISPR/Cas9 technology in aquaculture can significantly accelerate genetic improvement. Through CRISPR/Cas9, favorable gene variations can be rapidly introduced to enhance the growth rate and disease resistance of *M. rosenbergii*. Its efficiency and precision make it possible to achieve desired trait improvements in a short time [82]. Additionally, CRISPR technology can enhance resistance to environmental stresses, which is crucial as climate change poses increasing challenges to aquaculture. By precisely modifying the genome of *M. rosenbergii*, this technology can improve its adaptability to adverse conditions, thereby boosting yield and quality [83]. Furthermore, CRISPR technology can improve reproductive performance by editing genes related to reproduction, thus accelerating population expansion and meeting market demands more effectively [84]. The identification of genes related to growth retardation in *M. rosenbergii*, such as those identified through proteomic analysis of “iron prawn”, could potentially be corrected using PE. By precisely modifying the relevant genes, it may be possible to improve the growth performance of the prawn. In practice, CRISPR technology stands out for its ease of operation, low cost, and ability to perform multiple edits simultaneously, allowing for the rapid improvement of complex traits [85,86]. In summary, CRISPR genome editing technology holds great promise for *M. rosenbergii* breeding. It can comprehensively enhance the growth rate, disease resistance, and environmental adaptability, thereby promoting the sustainable development of the aquaculture industry [87].

## 7. Genome Editing for Enhanced Disease Resistance in *M. rosenbergii*

*M. rosenbergii* is susceptible to various diseases during aquaculture, which seriously affects yield and quality. CRISPR genome editing technology offers an effective way to enhance disease resistance in *M. rosenbergii*. In recent years, this technology has made significant progress in multiple fields, especially in improving disease resistance in plants and animals [88,89,90]. By using the CRISPR/Cas9 system, researchers can precisely edit specific genes to enhance an organism’s resistance to pathogens. By editing genes related to disease resistance, the resistance of *M. rosenbergii* to specific pathogens can be improved. Hemocyanin, a crucial protein in *M. rosenbergii*, has been studied in relation to its immune function. For instance, the cDNA of hemocyanin isoform 2 (MrHc2) was isolated and characterized. It contains three domains and plays an important role in the innate immune response to *M. rosenbergii* nodavirus (MrNV) [91]. Understanding the genetic basis of such immune-related proteins can potentially be manipulated using CRISPR–Cas nucleases to enhance the prawn’s immune capabilities. Another aspect is the study of cell surface receptors. The syndecan receptor gene (MrSDC) from *M. rosenbergii* was identified, and its function during bacterial infections was analyzed. MrSDC is expressed in various tissues and can bind to bacteria. Inhibition or overexpression of MrSDC affects the number of Aeromonas hydrophila in the hepatopancreas [92]. CRISPR–Cas nucleases could potentially be used to modify the expression or function of such receptor genes, providing new ways to combat bacterial infections in *M. rosenbergii*. BE and PE offers precise modification of single nucleotides in the genome of *M. rosenbergii*. In the context of disease resistance, studies have shown that certain genes play crucial roles in the prawn’s immune response. For example, the lipopolysaccharide- and beta-1,3-glucan-binding protein (LGBP) cDNA was cloned from *M. rosenbergii*. Its transcription was related to foreign material injection and the molt stage [93]. BE and PE could potentially be used to modify the *LGBP* gene to enhance the prawn’s immune response against pathogens. The identification of genes involved in the immune response, such as the thiol dependent peroxiredoxin gene *MrPrdx* [94], provides a potential target for BE to enhance the prawn’s antioxidant and immune capabilities. *MrPrdx* is highly expressed in various tissues and its expression is up-regulated after IHHNV infection. BE could potentially be used to modify the gene to increase its expression or improve its function.

Additionally, PE could be used to introduce beneficial mutations in genes involved in disease resistance, similar to how it has been proposed for treating β-thalassemia in humans [95], thereby enhancing the prawn’s ability to withstand pathogen infections. Using gene editing to enhance disease resistance can help reduce the harm of diseases to the *M. rosenbergii* farming industry, decrease drug use during the farming process, and achieve green and sustainable development.

## 8. Genome Editing for Sex Control in *M. rosenbergii* Breeding

The sex of *M. rosenbergii* significantly impacts its growth rate and economic value, with males typically growing faster and larger. Using CRISPR genome editing technology for sex control is crucial for enhancing farming efficiency. Research has found that certain genes play a key role in the sex determination and differentiation of *M. rosenbergii*. The study of genes related to sexual differentiation is also significant. The cloning and characterization of the insulin-like androgenic gland hormone binding protein (*MrIAGBP*) from *M. rosenbergii* revealed its role in IAG signaling [96]. The expression of *MrIAGBP*, *MrIAG*, and *MrDmrt11E* [97] was found to be related to the development of the androgenic gland, with the levels of both genes peaking at the adult stage [98]. Editing these specific genes can induce sex reversal during embryonic development, turning females into males and increasing the male population. In addition, the study of microRNAs (miRNAs) during gonadal development in *M. rosenbergii* has identified 1954 known and 129 novel miRNAs. A total of 41 miRNAs showed sex-biased expression patterns, and their putative target genes were enriched in reproduction related pathways [99]. BE and PE could be applied to modify the regulatory regions of these miRNAs or their target genes, potentially influencing the reproductive processes of *M. rosenbergii*, such as improving the breeding efficiency or controlling sexual development. The construction of a genomic bacterial artificial chromosome (BAC) library for *M. rosenbergii* and the initial analysis of ZW chromosome derived BAC inserts [100] offer opportunities. The identification of sex-linked genes on ZW chromosomes can be further explored using PE to manipulate the sexual development of *M. rosenbergii*. This could be crucial for the production of monosex populations, which is beneficial for aquaculture. This sex control technology offers new breeding strategies for the *M. rosenbergii* farming industry, helping to optimize the farm population structure and enhance economic returns.

## 9. Opportunities and Challenges in Genome Editing of *M. rosenbergii*

CRISPR–Cas9, base editors, and prime editors offer transformative tools to enhance traits like disease resistance and growth efficiency of *M. rosenbergii*. However, the species’ highly repetitive genome complicates precise editing, often resulting in suboptimal efficiency and off-target effects. Complementing these challenges are limitations in delivery systems critical for introducing editors into target tissues. While microinjection and electroporation remain primary methods, their efficacy in *M. rosenbergii* is constrained by biological barriers, such as the hard exoskeleton and embryonic accessibility. Recent innovations, including lipid-based nanoparticles and engineered ribonucleoprotein (RNP) complexes, aim to improve delivery precision and reduce toxicity. Integrating advanced editing tools with optimized delivery strategies holds the key to unlocking scalable, species-specific genetic improvements. As research bridges these technological gaps, the synergy between CRISPR-derived systems and tailored delivery platforms promises to revolutionize aquaculture breeding, paving the way for sustainable and resilient *M. rosenbergii* populations while addressing global food security demands.

Despite its advantages, the application of CRISPR in *M. rosenbergii* faces several species-specific genomic challenges. The species possesses a colossal genome (3.73 Gb) with over 60% repetitive elements, including simple sequence repeats (SSRs) and retrotransposons, which contribute to high off-target rates, up to 22% when editing the growth-related gene MrGHR due to noncoding sequence homology [101]. Polyploidy further complicates gene editing; for example, the presence of four MrHSP70 paralogs requires simultaneous multi-gene targeting to avoid functional redundancy [102]. In addition, extensive alternative splicing, with 2041 events identified, diminishes the editing efficacy. A case in point is the MrSDC gene, where targeting alternatively spliced exons led to incomplete disease resistance in 50% of edited individuals. Epigenetic factors also pose significant barriers. The CpG island in the promoter region exhibits a methylation level of 35%—more than twice that observed in mammals [103], which impairs Cas9 binding and reduces the homology-directed repair (HDR) efficiency to just 1.2%. In contrast, the non-homologous end-joining (NHEJ) repair rate at the MrIAG locus reaches 38% [104].

Beyond technical challenges, the considerations of ecology, public health, and ethics critically shape the commercialization of genome editing. The ecological risks from escape and unintended environmental release cannot be ignored. The gene-edited *M. rosenbergii* carrying dominant alleles such as fast-growth and pathogen-resistance could shift wild-population allele frequencies, thus causing irreversible genetic introgression [105]. Gene-edited *M. rosenbergii* escapes could cause biodiversity loss and ecosystem degradation through both direct competition or predation and indirect trophic cascades [106]. Furthermore, the off-target effects in *M. rosenbergii* may create novel allergens, posing a huge public health risk [107]. It is obliged to conduct more comprehensive food safety assessment and regulation. However, the opacity of labeling and information and traceability failure would result in consumer exposure without informed consent, triggering a crisis of confidence [108]. Meanwhile, key technology may be monopolized by a few companies, exacerbating resource inequality and forcing traditional farmers to rely on high-cost gene-edited seed stock. In addition, gene editing on *M. rosenbergii* may be protested by animal protection groups and cause cultural conflict as well. Therefore, advancing genome editing in *M. rosenbergii* demands a comprehensive strategy that integrates biological precision (species-specific editing tools), ecological safeguards (effective containment systems), and regulatory alignment. Together, these three pillars are essential for translating CRISPR’s potential into sustainable innovation in aquaculture.

## 10. Optimizing Editor Efficiency and Reducing Off-Targets

Although CRISPR–Cas9 technologies have achieved some progress in aquatic livestock [109,110,111,112], editing efficiency remains suboptimal, and off-target effects continue to hinder broader applications. To optimize editor efficiency, researchers are engineering key components, such as the Cas9 protein and deaminases, to improve their DNA-binding affinity and catalytic activity. For instance, structural optimization of the Cas9 protein has led to significant improvements in the editing efficiency [113]. Concurrently, refining gRNA design algorithms to enhance the specificity of gRNA-target sequence binding has effectively reduced off-target rates [114]. Guided engineering of base editor architectures has also successfully minimized unintended RNA editing activity while preserving targeted DNA editing efficacy [32,115,116]. Furthermore, the development of novel editing technologies or combinatorial approaches holds promise for simultaneously boosting the editing efficiency and mitigating off-target risks, thereby enhancing the precision and reliability of genome editing in *M. rosenbergii*. In summary, with ongoing advancements in CRISPR–Cas systems and their derivative tools, base editors and prime editors offer vast potential for applications in *M. rosenbergii*. These optimized tools not only elevate the efficiency and accuracy of genome editing but also unlock innovative possibilities for aquaculture and biotechnology, paving the way for transformative applications in these fields.

## 11. Development of Delivery Technologies for *M. rosenbergii* Genome Editing

The safe and efficient delivery of tools into target cells remains a significant challenge in CRISPR-based gene editing applications (Table 3). Current delivery methods for gene editing tools in *M. rosenbergii* primarily rely on microinjection, which suffers from operational complexity, low efficiency, and substantial embryo damage. The development of non-microinjection delivery technologies is critical to achieving efficient gene editing. Non-microinjection approaches include viral vectors (e.g., lentivirus [117] and adeno-associated virus [118]), engineered materials (e.g., liposomes [119], nanoparticles [120]), and extracellular vesicles, which are commonly used for delivering gene editing components in mammals. These systems offer advantages such as biocompatibility, high modifiability, and the ability to encapsulate editing tools for intracellular delivery with minimal cellular harm. However, challenges persist in the targeting specificity, biosafety, and scalable production of these carriers, necessitating further research and optimization. Physical transfection methods, such as electroporation, also show promise in non-microinjection delivery by overcoming limitations of traditional techniques and improving CRISPR component delivery efficiency [121]. The advancement of non-microinjection delivery technologies opens new possibilities for gene editing in *M. rosenbergii* and other invertebrates. By integrating efficient polymer-based carriers, physical transfection methods, and engineered materials, researchers aim to achieve the safe and highly efficient delivery of gene editing tools in *M. rosenbergii*, thereby accelerating both fundamental research and practical applications in aquaculture biotechnology.

## 12. Functional Gene Mining and Multi-Gene Editing in *M. rosenbergii*

The mechanisms of sex differentiation and growth regulation in *M. rosenbergii* are not yet fully understood, which limits its potential in aquaculture. The rapid advancement of genome editing technologies offers new opportunities for functional gene mining [122] and multi-gene editing [123,124,125]. The progress in genome editing technologies has made multi-gene editing possible in *M. rosenbergii*. Using genome editing tools, the simultaneous editing of multiple target genes or high-throughput gene editing can be achieved, thereby accelerating the validation and application of functional genes, such as key genes related to sex differentiation. Additionally, genome editing technologies can be used to create gene mutants with specific traits, offering new strategies for the breeding and improvement of *M. rosenbergii*. Finally, the application of genome editing technologies in *M. rosenbergii* is not limited to sex differentiation and growth regulation but can also be extended to research on other important traits such as disease resistance and environmental adaptability. By continuously optimizing genome editing tools and developing novel editing strategies, researchers can more precisely manipulate the genome of *M. rosenbergii*, achieving comprehensive genetic improvement. Multi-gene editing faces challenges such as the editing efficiency, the complexity of gene interactions, and an increased risk of off-target effects. Further optimization of editing strategies and technical approaches is needed to achieve efficient and precise multi-gene editing. The functional gene mining and multi-gene editing of *M. rosenbergii* are of great significance for gaining in-depth insights into its biological characteristics and genetic improvement.

## 13. Conclusions

The application of technologies such as CRISPR–Cas9, base editors, and prime editors has revolutionized the field of genome editing, offering unprecedented precision and versatility in modifying genetic material. CRISPR–Cas9, derived from a microbial adaptive immune system, allows for targeted DNA double-strand breaks, which can be repaired to introduce specific genetic changes. This system has been widely adopted due to its simplicity and efficiency compared to earlier genome editing technologies like Zinc-finger nucleases (ZFNs) [126] and transcription activator-like (TAL) effector nucleases (TALENs) [127]. However, the introduction of double-strand breaks can lead to unintended large genomic deletions, which pose challenges for certain applications. To address these limitations, base editors and prime editors have been developed as alternatives that do not require double-strand breaks. Base editors enable precise nucleotide conversions, such as C-to-T or A-to-G, by utilizing deaminase enzymes fused to a catalytically impaired Cas9. These editors have been further refined to expand their targeting scope and reduce off-target effects, making them powerful tools for both research and therapeutic applications. Prime editors, on the other hand, offer even greater flexibility by allowing for a wider range of genetic modifications, including insertions and deletions, without the need for donor DNA or double-strand breaks. The development of efficient delivery methods for these genome editing tools is crucial for their successful application in various organisms. Non-microinjection delivery systems, such as those utilizing ribonucleoprotein complexes or synthetic carriers, have been explored to enhance the efficiency and reduce the off-target effects of CRISPR/Cas9 systems. These methods offer advantages such as transient genome editing and improved delivery to target cells, which are essential for advancing therapeutic applications. As these technologies continue to evolve, they hold great promise for advancing our understanding of genetic diseases and developing novel treatments.

In summary, the application of technologies such as CRISPR–Cas9, base editors, and prime editors, along with the development of efficient non-microinjection delivery systems, will provide robust technical support for the gene editing-based breeding of *M. rosenbergii*. This has the potential to cultivate new strains of *M. rosenbergii* with superior traits and will promote the development of the aquaculture industry.

## Figures and Tables

**Table 1:** Summary of key base editors.

Base Editors	Editing Types	Editor Structures	Editing Efficiency	Windows (bp)	PAMs	Ref.
BE1	C > T	APOBEC1-dCas9 (D10A, H840A)	~7.7%	4~8	NGG	[19]
BE2	C > T	APOBEC1-dCas9 (D10A, H840A)-UGI-NLS	~20%	4~8	NGG	[19]
BE3	C > T	APOBEC1-nCas9 (D10A)-UGI-NLS	~37%	4~8	NGG	[19]
BE4	C > T	APOBEC1-nCas9 (D10A)-UGI-UGI-NLS	~50%	4~8	NGG	[19]
BE4max	C > T	NLS-APOBEC1-nCas9 (D10A)-UGI-UGI-NLS	~70%	4~8	NGG	[32]
FNLS	C > T	FNLS-APOBEC1-nCas9 (D10A)-UGI-NLS	~75%	4~8	NGG	[33]
AncBE4max	C > T	NLS-APOBEC-nCas9 (D10A)-UGI-UGI-NLS	~75%	4~8	NGG	[32]
BE4-Gam	C > T	Gam-APOBEC1-nCas9 (D10A)-UGI-UGI	~50%	4~8	NGG	[34]
YE1-BE3	C > T	APOBEC1 (W90Y R126E)-nCas9 (D10A)-UGI	~50%	4~7	NGG	[35]
EE-BE3	C > T	APOBEC1 (R126E R132E)-nCas9 (D10A)-UGI	~40%	5~6	NGG	[35]
YE2-BE3	C > T	APOBEC1 (W90Y R132E)-nCas9 (D10A)-UGI	~40%	5~6	NGG	[35]
YEE-BE3	C > T	APOBEC1 (W90Y R126E R132E)-nCas9 (D10A)-UGI	~30%	5~6	NGG	[35]
VQR-BE3	C > T	APOBEC1-VQR nCas9 (D10A)-UGI	~40%	4~11	NGAN	[35]
VRER-BE3	C > T	APOBEC1-VRER nCas9 (D10A)-UGI	~40%	3~10	NGCG	[35]
SaBE3	C > T	APOBEC1-nSaCas9 (D10A)-UGI	~50%	3~12	NNGRRT	[35]
SaBE4	C > T	APOBEC1-nSaCas9 (D10A)-UGI-UGI	~50%	3~12	NNGRRT	[34]
SaBE4-Gam	C > T	Gam-APOBEC1-nSaCas9 (D10A)-UGI-UGI	~60%	3~12	NNGRRT	[34]
Sa (KKH)-BE3	C > T	APOBEC1-KKH nSaCas9 (D10A)-UGI	~50%	3~12	NNNRRT	[34]
xBE3	C > T	APOBEC1-nXCas9 (D10A)-UGI	~37%	4~8	NG	[36]
eA3A-BE3	C > T	APOBEC3A N57G-nCas9 (D10A)-UGI	~55%	4~8	NGG	[37]
A3A-BE3	C > T	hAPOBEC3A-nCas9 (D10A)-UGI	~50%	4~8	NGG	[38]
AID	C > T	nCas9 (D10A)-CDA1-UGI	~35%	2~4	NGG	[39]
AID-NG	C > T	nCas9 (D10A)-NG-CDA1-UGI	~35%	2~4	NG	[40]
BE-PLUS	C > T	10×GCN4-nCas9 (D10A), scFv-rAPOBEC1-UGI	~40%	4~14	NGG	[41]
SECURE BE3-AA	C > T	APOBEC1 (R33A K34A)-nCas9 (D10A)-UGI	~30%	4~8	NGG	[22]
BE-PIGS	C > T	nCas9 N-APOBEC1-nCas9 C-UGI-UGI-NLS	~40%	7~13	NGG	[42]
eTd-CBE	C > T	TadA8e (N46L)-nCas9 (D10A)-P2A-UGI-UGI-NLS	~80%	6~7	NGG	[43]
TadCBEd	C > T	NLS-TadA CD-nCas9 (D10A)-UGI-UGI-NLS	~70%	4~8	NGG	[44]
aTdCBE	C > T	NLS-AjTadA.v2-nCas9 (D10A)-UGI-UGI-NLS	~70%	4~8	NGG	[45]
ABE7.10	A > G	TadA-TadA mutant-nCas9 (D10A)	~60%	4~7	NGG	[46]
miniABE7.10	A >G	TadA mutant-nCas9 (D10A)	~70%	4~7	NGG	[46]
ABEmax	A > G	NLS-TadA-TadA mutant-nCas9 (D10A)-NLS	~80%	4~7	NGG	[21]
PABE7	A > G	TadA-TadA mutant-nCas9 (D10A)-3xNLS	~35%	4~7	NGG	[47]
ABE8	A > G	TadA-TadA mutant-nCas9 (D10A)-NLS	~80%	4~8	NGG	[48]
ABE8e	A > G	NLS-TadA mutant 8e-nCas9 (D10A)-NLS	~80%	4~8	NGG	[49]
SpRY-ABE8e^F148A^	A > G	nSpRY-TadA*^F148A^-NLS	~80%	4–7	PAMless	[50]
xABE	A > G	TadA-TadA mutant-n XCas9 (D10A)	~70%	4~7	NG	[51]
ABESa	A > G	TadA-TadA mutant-nSaCas9 (D10A)	~45%	6~12	NNGRRT	[51]
VQR-ABE	A > G	TadA-TadA mutant-VQR nCas9 (D10A)	~70%	4~6	NGA	[51]
SECURE-ABE	A > G	NLS-TadA mutant-nCas9 (D10A)-NLS	~60%	4~7	NGG	[52]
ABE9	A > G	NLS-TadA8e-N108Q/L145T-nCas9 (D10A)-NLS	~70%	5~6	NGG	[53]
hyABE	A > G	NLS-TadA 8e-Rad51DBD-nCas9 (D10A)-NLS	~80%	4~8, 10~15	NGG	[54]
STEME-1	C > T, A > G	A3A-TadA-TadA mutant7.10-nCas9 (D10A)-NLS-UGI-NLS	~15%	4~8	NGG	[55]
Target-ACEmax	C > T, A > G	NLS-TadA-TadA mutant7.10-nCas9 (D10A)-CDA1-NLS-UGI	~20%	4~8	NGG	[56]
SPACE	C > T, A > G	TadA mutant V82G-nCas9 (D10A)-CDA1-UGI-UGI	~15%	4~8	NGG	[57]
A&C-BEmax	C > T, A > G	NLS-AID-TadA-TadA mutant-nCas9 (D10A)-UGI-UGI-NLS	~15%	4~8, 1~13	NGG	[58]
miniAGBE-4	C > G/A/T, A > G	NLS-A3Ai-TadA8e (V106W)-nCas9 (D10A)-NLS	~30%	4~8	NGG	[59]
PhieDBE	C > T, A > G	NLS-evoFERNY-TadA 8e-Rad51DBD-nSpGCas9 (D10A)-UGI-UGI-NLS	~40%	5~9, 5~8	NG	[60]
BDBE	C > G/A/T, A > G/C/T	NLS-TadA mutant dual-nCas9 (D10A)-AAG mutant-NLS	~35%	4~8	NGG	[61]
CGBE	C > G	APOBEC1-nCas9 (D10A)-UNG-NLS	~25%	5~6	NGG	[62]
Td-CGBE	C > G	NLS-TadA8e (N46L)-nCas9 (D10A)-NLS	~30%	5~6	NGG	[43]
AID-nCas9-UNG	C > A	AID-nCas9 (D10A)-UNG	~80%	4~8	NGG	[63]
DAF-CBE	C > G	CDG-nCas9 (D10A)	~10%	2~6	NGG	[64]
gCBEv2	C > G	NLS-UNG mutant-nCas9 (D10A)-NLS	~70%	2~6	NGG	[28]
ACBE-Q	A > C	NLS-nCas9 N-AAG mutant-TadA8e-nCas9 C-NLS	~27%	4~6	NGG	[30]
AXBEv2	A > C/T	NLS-TadA8e-nCas9 (D10A)-AAG mutant EF-NLS	~60%	7~9	NGG	[30]
AYBEv3	A > C/T	NLS-TadA8e-nCas9 (D10A)-NLS-AAG mutant-NLS	~70%	7~8	NGG	[31]
gGBEv6.3	G > C/T	NLS-nCas9-NLS-AAG mutant-NLS	~70%	6~11	NGG	[27]
DAF-TBE	T > G	TDG-nCas9 (D10A)	~10%	2~6	NGG	[65]
gTBEv3	T > C/G	NLS-UNG mutant-nCas9 (D10A)-NLS	~70%	3~7	NGG	[28]
TBE	T > C/G	UNG mutant-nCas9 (D10A)	~40%	3~8	NGG	[29]

**Table 2:** Summary of key prime editors.

Prime Editors	Editor Structures	Guide RNAs	Editing Efficiency	PAMs	Ref.
PE1	NLS-nCas9 (H840A)-MMLV RT-NLS	pegRNA	~5–20%	NGG	[6]
PE2	NLS-nCas9 (H840A)-MMLV RT mutant-NLS	pegRNA	~10–30%	NGG	[6]
PE2*	NLS-NLS-nCas9 (H840A)-MMLV RT mutant-NLS-NLS	pegRNA	~15–40%	NGG	[73]
PE3	NLS-nCas9 (H840A)-MMLV RT mutant-NLS	pegRNA+nick-sgRNA	~20–50%	NGG	[6]
PE3b	NLS-nCas9 (H840A)-MMLV RT mutant-NLS	pegRNA+optimized nick-sgRNA	~25–55%	NGG	[74]
sPE	NLS-Sp nCas9 (H840A)-NLS + NLS-MMLV RT mutant-NLS	pegRNA+nick-sgRNA	~20–50%	NGG	[75]
ePE	NLS-Sp nCas9 (H840A)-NLS + NLS-MMLV RT mutant-NLS	pegRNA+nick-sgRNA	~20–50%	NGG	[76]
PE4	NLS-Sp nCas9 (H840A)-MMLV RT mutant-NLS + MLH1dn	pegRNA	~30–60%	NG	[7]
PE5	NLS-Sp nCas9 (H840A)-MMLV RT mutant-NLS + MLH1dn	pegRNA+nick-sgRNA	~40–70%	NG	[7]
PE6	NLS-Sp nCas9 (H840A)-MMLV RT mutant (K538M/D583N)-NLS + MLH1dn	pegRNA+nick-sgRNA	~30–75%	NG	[8]
PEn	Cas9-MMLV RT-NLS	pegRNA	~15–40%	NGG	[77]
tPE	NLS-nCas9 (H840A)-MMLV RT mutant-NLS	tRNA scaffold-modified pegRNA+nick-sgRNA	~25–60%	NGG	[78]
Dual PE	NLS-nCas9 (H840A)-MMLV RT mutant-NLS	Dual pegRNAs+nick-sgRNA	~50–80%	NGG	[79]
PEmax	NLS-Sp nCas9 (R221K N394K H840A)-NLS-MMLV RT mutant-NLS-NLS	epegRNA+nick-sgRNA	~60–80%	NGG/ NRN	[7]
PE7	NLS-Sp nCas9 (H840A)-MMLV RT mutant-NLS + MLH1dn (Engineered MMR-deficient background)	epegRNA+nick-sgRNA	~50–85%	NG/ NRN	[9]

**Table 3:** The summary of CRISPR or drug delivery systems.

Delivery Types	Mechanisms	Advantages	Challenges
Nanoparticles	Polymeric/lipid-based carriers for targeted delivery.	High stability, controlled release, biocompatibility.	Potential toxicity, complex synthesis.
Liposomes	Lipid bilayer vesicles encapsulating drugs.	Enhanced solubility, reduced systemic toxicity.	Short shelf-life, leakage of payload.
Hydrogels	Crosslinked polymer networks for sustained release.	Tunable porosity, stimuli-responsive release.	Limited drug-loading capacity.
Microneedles	Painless transdermal patches with micron-sized needles.	Non-invasive, bypasses first-pass metabolism.	Limited drug capacity, skin irritation.
Dendrimers	Branched polymers with high surface functionality.	Precise size control, multifunctional drug loading.	Toxicity concerns, scalability issues.
Viral vectors	Engineered viruses (e.g., adenovirus, lentivirus) for gene delivery.	High transfection efficiency.	Immunogenicity, insertional mutagenesis risks.
Exosomes	Natural extracellular vesicles for cell-to-cell communication.	Biocompatible, low immunogenicity.	Isolation/purification challenges.
Cell-based systems	Engineered cells (e.g., RBCs, stem cells) as carriers.	Prolonged circulation, inherent targeting.	Complex manufacturing, regulatory hurdles.
VLPs (Virus-like particles)	Self-assembled viral protein structures mimicking viruses (non-infectious).	High biocompatibility, efficient cellular uptake, tunable surface modifications.	Complex production, potential pre-existing immunity, scalability limitations.
Electroporation	Electric pulses create transient pores in cell membranes for cargo delivery.	Rapid and efficient delivery, works for large molecules (e.g., plasmids).	Cell/tissue damage risk, limited to accessible tissues, variable efficiency.

## Data Availability

Not applicable.
